# Atherogenic Dyslipidemia in Children and Adolescents: Current Evidence, Clinical Challenges, and Future Perspectives

**DOI:** 10.3390/jcdd13020089

**Published:** 2026-02-11

**Authors:** Marco Giussani, Manuela Casula, Antonina Orlando, Gianfranco Parati, Simonetta Genovesi

**Affiliations:** 1 Istituto Auxologico Italiano, Istituto Ricovero Cura Carattere Scientifico (IRCCS), 20135 Milan, Italy; m.giussani@auxologico.it (M.G.); a.orlando@auxologico.it (A.O.); parati@auxologico.it (G.P.); 2 IRCCS MultiMedica, 20099 Sesto San Giovanni, Italy; manuela.casula@unimi.it; 3 Epidemiology and Preventive Pharmacology Service (SEFAP), Department of Pharmacological and Biomolecular Sciences, University of Milan, 20133 Milan, Italy; 4 Department of Medicine and Surgery, University of Milano-Bicocca, 20126 Milan, Italy

**Keywords:** adolescents, atherosclerosis, cardiovascular disease, children, dyslipidemia

## Abstract

Atherogenic dyslipidemia is a condition characterized by high lipid levels that promote the development of atherosclerosis. While the clinical manifestations of atherosclerosis typically manifest in adulthood, early vascular damage can be identified in children and adolescents. Dyslipidemia is not uncommon in childhood and adolescence, and its development depends on the interaction between genetic and environmental factors. Forms caused by genetic defects tend to manifest earlier and usually require drug treatment. Forms caused by unhealthy lifestyles and eating habits tend to manifest later and often only require dietary and behavioural treatment. The review describes the most common primary forms, diagnostic criteria and treatment options, both pharmacological and non-pharmacological, emphasizing the differences and specificities of dyslipidemia in children compared to adults. The review’s objective is also to provide a clinically focused summary of the current evidence on atherogenic dyslipidemia in children and adolescents.

## 1. Introduction

**Figure 1:**
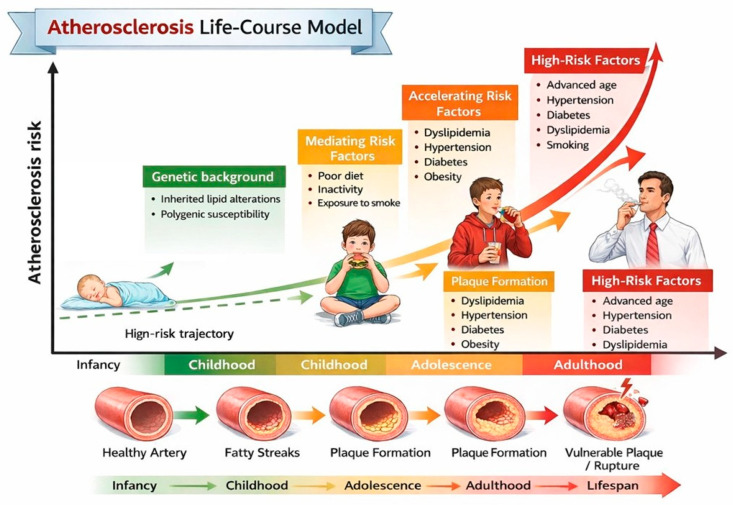
Atherosclerosis life-course model. This schematic shows how atherosclerosis develops progressively from early childhood to adulthood.

Atherogenic dyslipidemia is one of the earliest and most significant modifiable determinants of lifelong cardiovascular (CV) risk [1]. Although clinical CV events typically occur decades later, there is substantial evidence indicating that atherosclerotic processes begin in childhood and progress silently over time, particularly when lipid abnormalities are persistent (Figure 1) [2]. Almost half of children with obesity show an abnormal lipid profile, characterized by increased low-density lipoprotein (LDL) cholesterol and triglyceride (TG) values and decreased high-density lipoprotein (HDL) cholesterol values [3]. Evidence from imaging studies indicates that early lipid abnormalities in children with heterozygous familial hypercholesterolemia (HeFH) and those with familial combined hyperlipidemia (FCHL) are associated with subclinical vascular changes such as increased carotid intima-media thickness and endothelial dysfunction [4]. Recognizing these lipid patterns in children is therefore crucial, not only for accurate risk stratification but also for timely implementation of preventive strategies, including lifestyle modification and, where indicated, pharmacologic intervention. Early intervention can alter the trajectory of cardiovascular risk, potentially delaying or preventing the onset of clinically significant atherosclerosis in adulthood [5].

However, dyslipidemia in children remains frequently underdiagnosed and undertreated, partly due to heterogeneous screening strategies, evolving therapeutic options, and persistent concerns about long-term pharmacological treatment [6,7]. In this context, it is essential to promote knowledge and awareness among physicians, especially pediatricians and primary care providers, to ensure timely diagnosis, adequate risk stratification and evidence-based management. The aim of this review is to provide a clinically oriented synthesis of the current evidence on atherogenic dyslipidemia in children and adolescents.

## 2. Definition of Dyslipidemia

Dyslipidemia is defined as a deviation of plasma lipid levels from their normal values [8].

Table 1 shows the reference threshold values used to define normal plasma lipid concentrations in children and adolescents. These references are adopted by all guidelines and are therefore mandatory for determining non-physiological ranges. However, the table oversimplifies the situation, as plasma lipid values are not constant from birth to adolescence and adulthood, and are influenced by age, gender and pubertal development. Some authors have proposed reference percentiles according to age and gender. One US study, involving a sample of 6067, 12–19-year-olds adolescents, provided percentiles for total cholesterol, LDL cholesterol, HDL cholesterol and TGs [9]. A second study considered 8071 children and adolescents (3823 boys and 4248 girls) aged 8–18 years, without reporting TG values [10]. Both studies demonstrate a decrease in total and LDL cholesterol values at the beginning of puberty, followed by an increase once development is complete. Since puberty begins earlier in girls than in boys, the reference percentiles for the two genders take this into account. There are no precise references for children under the age of eight, but a study has been published which aimed to compare total and LDL cholesterol values in children with FH using neonatal screening [11]. At birth, children without FH had average total and LDL cholesterol values of 74.6 and 26.3 mg/dL, respectively. Children with FH, on the other hand, had values of 94.7 and 47.2 mg/dL respectively. By the age of three, healthy children had LDL cholesterol values of 91.6 mg/dL, whereas children with FH had values of 215 mg/dL. Although the comparison of LDL -cholesterol between FH and non-FH subjects shows a significant difference from birth onwards, there is significant overlap in values. This makes diagnosing FH in this age group based solely on plasma cholesterol values unreliable. Furthermore, a significant proportion of children carrying a pathogenic heterozygous FH mutation have normal total and LDL cholesterol values for much of their childhood, only showing pathological values at a later stage. Therefore, careful follow-up is recommended for children of parents with FH, even if their cholesterol levels are normal in the early years of life [12]. Many dyslipidemias are atherogenic, meaning they promote the deposition of cholesterol in artery walls, leading to the formation of atherosclerotic plaque. If left untreated during childhood, dyslipidemia can result in prolonged exposure to atherogenic lipoproteins and accelerate the atherosclerotic process [13]. Accordingly, the condition may manifest very early, with the first atherosclerotic lesions of the arterial wall (lipid striae) detectable in the first or second decade of life in developed countries [14,15]. The rate at which atherosclerosis progresses depends on the presence of risk factors, their severity, and the length of exposure [16].

Lipid metabolism in children follows the same pathways as in adults; however, some specific characteristics and peculiarities in the clinical manifestations and management of diseases in pediatrics must be carefully considered. To better understand these aspects, knowledge of the main aspects of lipid metabolism and transport in the body is necessary.

## 3. Lipid Metabolism and Transport

**Figure 2:**
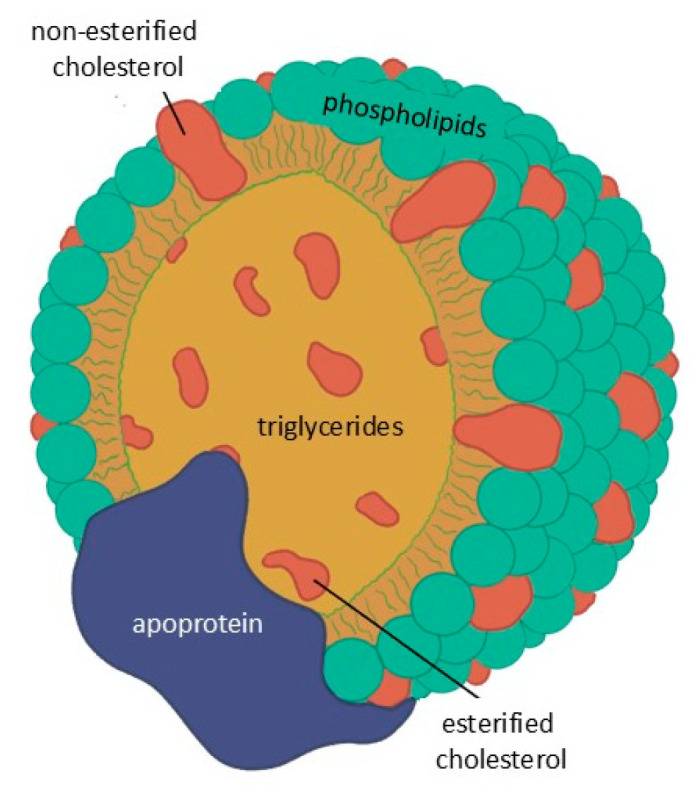
Basic structure common to all lipoproteins.

Lipids are not water-soluble and circulate in the blood as lipoproteins, with the exception of free fatty acids which are transported by albumin. These particles consist of a core of TGs and esterified cholesterol surrounded by phospholipids and free cholesterol, with surface apo-lipoproteins that mediate structural stability, enzymatic activity, and receptor interactions (Figure 2).

**Figure 3:**
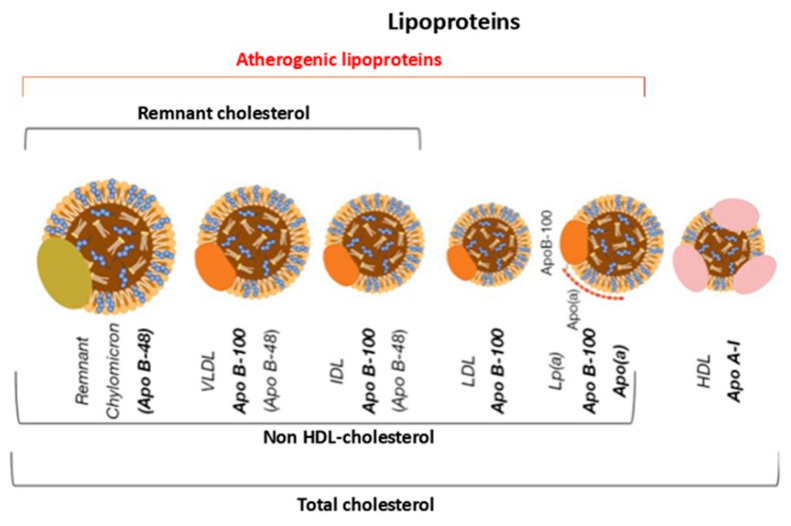
Graphical representation of the main types of lipoproteins. Apo B-48 = apoprotein B-48; Apo B-100 = apoprotein B-100; Lp(a) = Lipoprotein(a); Apo A-I = apoprotein A-I; VLDL = very low-density lipoprotein; IDL = intermediate density lipoprotein; LDL = low-density lipoprotein; HDL = high-density lipoprotein.

The size and apolipoprotein composition of lipoproteins differ substantially, as does their lipid content. These differences directly influence their metabolic behaviour, plasma residence time and atherogenic potential [17] (Figure 3).

**Figure 4:**
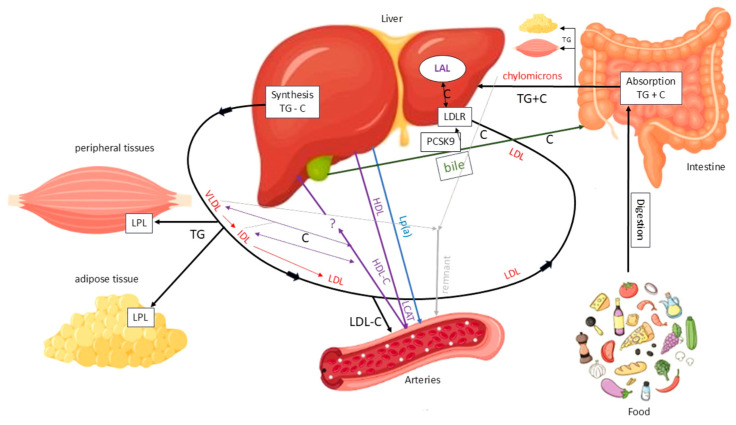
Main pathways of lipid metabolism and transport. C = cholesterol; LAL = lysosomal acid lipase; LDL = low-density lipoprotein; LDLR = low-density lipoprotein receptor; LPL = lipoprotein lipase; TG = triglyceride. Solid black arrows indicate the major pathways of TG and C transport between intestine, liver, peripheral tissues, and circulation. Red arrows indicate the conversion of VLDL to IDL and LDL particles. Purple arrows indicate HDL-mediated C efflux and transport to the liver. Blue arrows indicate Lp(a) mediated C transport from the liver to arteries. Green arrows indicate biliary C excretion. Grey arrows represent chylomicron remnants uptake by the liver.

Cholesterol originates from two sources: an exogenous source from dietary intake and an endogenous source mainly synthesized in the liver. Dietary TGs and cholesterol are absorbed in the intestine and transported to the liver via chylomicrons. The liver also produces TGs and cholesterol, which are secreted as very low-density lipoproteins (VLDLs), favouring TG/cholesterol ratio. In the bloodstream, VLDLs release TGs to peripheral tissues via lipoprotein lipase (LPL), after which they are progressively converted into intermediate-density lipoproteins (IDLs) with a balanced TG/cholesterol ratio. IDLs are then converted into LDLs, which are cholesterol-rich. The main function of this system is to deliver TGs to peripheral and adipose tissues for use or storage, while also transporting cholesterol, particularly via LDL, to the liver and other tissues (Figure 4).

**Figure 5:**
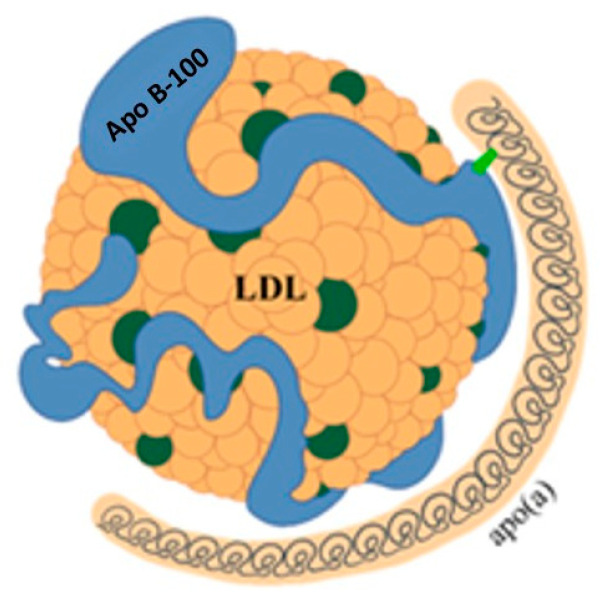
Structure of lipoprotein(a). LDL = low-density lipoprotein; Apo B-100 = apoprotein B-100; apo(a) = apoprotein(a).

LDLs are primarily removed from the bloodstream by the liver via LDL receptors (LDLRs), the levels of which are regulated by factors including proprotein convertase subtilisin/kexin type 9 (PCSK9). PCSK9 binds to the LDL–LDLR complex, promoting its lysosomal degradation and preventing receptor recycling. This reduces hepatic LDL clearance. Under normal conditions, the LDL-LDLR complex is internalized, the LDL particle is degraded by lysosomal acid lipase (LAL) and the receptor is recycled to the membrane. However, not all LDL particles follow this pathway. A fraction of them, particularly when plasma concentrations are elevated, can penetrate the arterial wall and promote atherosclerotic plaque formation by depositing cholesterol. Additionally, remnant lipoproteins from VLDL and chylomicron metabolism can accumulate in the vascular wall, carrying cholesterol and exerting a potentially atherogenic effect. Another lipoprotein that has been linked to atherosclerosis is lipoprotein(a) (Lp(a)) (Figure 5) [18].

While the physiological function of Lp(a) remains unclear, it is recognized as an important independent cardiovascular risk factor [18]. Lp(a) consists of a particle with characteristics similar to LDL, to which another protein called apolipoprotein(a) (apo(a)) is bound by disulphide bridges. Apo(a) is synthesized in the liver and consists of a series of subunits, called kringles. One of these can be repeated a variable number of times, from a few units up to 40. This variability results in significant heterogeneity of Lp(a) in terms of size and molecular weight, posing a problem for its measurement. Due to this variability, it is more accurate to express Lp(a) concentration in molar units (nmol/L). However, in clinical practice, the unit of mass (mg/dL) is still widely used. A conversion factor of between 2 and 2.5 has been suggested, but this estimate is subject to wide variability and therefore potential errors [18,19].

In addition to atherogenic lipoproteins, cholesterol transport also involves another class of lipoprotein, HDLs, which are synthesized mainly in the liver and intestine. HDLs have a lower lipid content than LDLs and a higher protein content. Unlike LDLs, HDLs actively participate in cardiovascular protection mechanisms by promoting the removal of excess cholesterol from peripheral tissues and arterial walls, as well as contributing to the modulation of local inflammation [20].

Assessing cardiovascular risk among atherogenic lipoproteins can be improved by measuring either apoB or non-HDL cholesterol. As each atherogenic lipoprotein particle contains only one apoB molecule, plasma apoB concentration directly reflects the total number of potentially atherogenic particles in the bloodstream [21]. Therefore, the apoB measurement provides an accurate indication of the overall atherogenic load, offering more insight than LDL cholesterol concentration alone. Non-HDL cholesterol, which is obtained by subtracting HDL cholesterol from total cholesterol, represents the total amount of cholesterol carried by potentially atherogenic lipoproteins, and it correlates well with apoB values [22].

## 4. Classification of Dyslipidemias

The classification of dyslipidemias is relatively simple and is based on alterations to the main lipid fractions. Isolated increases in cholesterol or TGs may be observed, or mixed forms characterized by an increase in both components. Potentially, atherogenic dyslipidemia may also include a reduction in HDL cholesterol and an increase in Lp(a) levels [23]. Dyslipidemia can be primary or secondary to pathological conditions or medication use. Table 2 describes the secondary forms of dyslipidemia, which are rare in children and adolescents. This review focuses solely on primary dyslipidemia, bearing in mind that the presence of a secondary form must always be considered and ruled out.

The etiopathogenesis of dyslipidemias results from the interaction between genetic and environmental factors, particularly lifestyle and dietary habits. Forms with a strong genetic basis typically present in early childhood, whereas those predominantly driven by environmental influences tend to manifest later, often in older children or adolescents, and are more amenable to dietary and behavioural interventions.

Although some dyslipidemias are common in childhood and others occur almost exclusively in adults, an understanding of adult forms remains essential when evaluating pediatric patients, particularly to assess family history and ensure an accurate diagnosis.

### 4.1. Isolated Hypercholesterolemia

Isolated hypercholesterolemia is common in children. It has been reported that around 4.5% of children in the 7–11 age group have total cholesterol levels above 200 mg/dL [24]. US data report much higher figures [25]. In most cases, this phenotype is an expression of FH or common polygenic hypercholesterolemia.

#### 4.1.1. Familial Hypercholesterolemia (FH)

**Figure 6:**
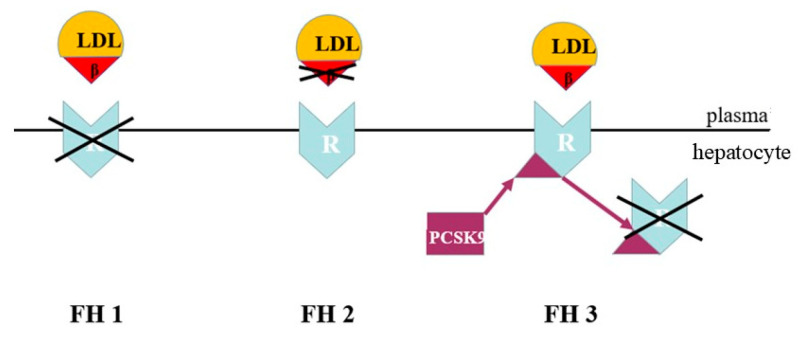
Genetic defects of co-dominant familial hypercholesterolemia. LDL = low-density lipoprotein; β = apo B-100 ligand; R = LDL cholesterol receptor; PCSK9 = proprotein convertase subtilisin/kexin type 9. FH1 = defect in the LDL cholesterol receptor. FH2 = defect in the LDL cholesterol apo B-100 ligand. FH3 = defect with gain of function in PCSK9 synthesis.

FH is a genetic form of dyslipidaemia, transmitted in an autosomal co-dominant manner. It is caused by defects in the mechanisms of hepatic LDL removal. In most cases, FH is due to variants of the LDLR gene, which result in reduced LDL uptake by the liver and a marked increase in LDL cholesterol in the bloodstream. In a minority of cases, FH is caused by variants of the APOB gene that alter the binding between LDL and the receptor. Alternatively, it can be caused by gain-of-function variants of the PCSK9 gene, which lead to increased receptor degradation and reduced LDL clearance [26] (Figure 6).

Although they involve different molecular mechanisms, all these alterations have the same functional effect: increased persistence of LDLs in the bloodstream. This is responsible for the high and persistent levels of LDL cholesterol that characterize FH.

FH is the most common genetic disorder of lipid metabolism. It affects a co-dominant gene and can occur in either the heterozygous (1 in 250–300 cases) or homozygous (1 in 350,000–450,000 cases) form [27]. From a genetic point of view, the distinction between HeFH and homozygous (HoFH) forms is not always clear-cut and must be interpreted in relation to the residual functionality of the alleles involved. In the heterozygous form, a pathogenic variant is present on one allele only, while the other allele retains normal or near-normal function. In such cases, LDL cholesterol levels can range from high to very high, depending on the residual activity of the variant-carrying allele. However, some heterozygous patients have loss-of-function variants and may present a clinical phenotype similar to the more severe forms. Affected individuals may present with biallelic forms, including cases where two pathogenic variants affect the same gene or, more rarely, variants in two distinct genes [28], often an alteration of *LDLR* associated with an alteration of the genes encoding apoB or CPSK9. Although the phenotype is influenced by the variants, biallelic forms generally result in extremely high levels of LDL cholesterol and a very high risk of early cardiovascular events, which can occur in the first or second decade of life. The clinical manifestations of FH mainly depend on the extent and duration of the increase in LDL cholesterol. The disease’s main hallmarks, such as xanthomas or xanthelasmas, are more prevalent in adults, whereas in children they only appear in the most severe cases.

#### 4.1.2. Autosomal Recessive Hypercholesterolemia

This form is very rare and clinically indistinguishable from HoFH. It is autosomal recessive, only manifesting in cases of homozygosity. It is caused by pathogenic variants in the *LDLRAP1* gene, which encodes an adaptor protein that promotes the internalization of LDLR after it has bound to lipoprotein. Consequently, affected individuals have very high cholesterol levels, whereas their parents, who are heterozygous carriers, have normal levels [29].

#### 4.1.3. Common Polygenic Hypercholesterolemia

Common polygenic hypercholesterolemia is the most prevalent form of hypercholesterolemia in children, although its prevalence is lower than in adults (1 in 50 cases) as it takes time to manifest. It is caused by several minor defects in cholesterol metabolism or transport coexisting together, and is associated with unhealthy lifestyles and diets [30]. Generally, this type of hypercholesterolemia is characterized by lower cholesterol levels than FH. However, in cases involving particularly unhealthy lifestyles and/or a large number of genes whose effects add up, cholesterol levels may be indistinguishable from those observed in the presence of HeFH.

#### 4.1.4. Phytosterolaemia (Sitosterolaemia)

Phytosterolaemia is an extremely rare genetic disorder, affecting one in a million people. It is characterized by excessive intestinal absorption and reduced biliary excretion of plant sterols, due to pathogenic variants in the ABCG5 and ABCG8 genes. These genes encode transporters responsible for eliminating the sterols [31]. As commonly used laboratory methods cannot distinguish between cholesterol and phytosterols, the condition may present as significant hypercholesterolemia. This is often resistant to statin therapy, but responds well to ezetimibe, a drug that reduces the absorption of phytosterols in the intestine. The disease begins in childhood or adolescence, presenting with early tendon xanthomas, hematological changes (particularly hemolysis or thrombocytopenia) and joint symptoms. Atherosclerotic lesions develop early, and cardiovascular events can occur from around the age of 40 if the condition remains undiagnosed and untreated.

#### 4.1.5. HyperLp(a)

As previously mentioned, Lp(a) is considered an independent risk factor for cardiovascular disease. It is estimated that Lp(a) has a significantly higher atherogenic potential than LDL. This is due to the cholesterol contained in the LDL-like portion of Lp(a) and the high proportion of oxidized phospholipids present, which stimulate low-intensity inflammatory processes in atherosclerosis [32]. Although Lp(a) is measured alongside other LDLs, their values are not necessarily correlated. It is possible to have high Lp(a) levels despite having normal cholesterol levels. The risk of developing cardiovascular disease increases with the plasma concentration of Lp(a). Individuals who produce larger apo(a) isoforms tend to have lower Lp(a) levels and are therefore at lower cardiovascular risk, whereas the opposite is true for those who produce apo(a) with a lower molecular weight. In addition to apo(a) size, an increased cardiovascular risk has been associated with specific *LPA* gene haplotypes. The quantity and characteristics of Lp(a) produced by each individual are mainly determined by the activity of their maternal and paternal *LPA* genes, and are scarcely influenced by environmental factors such as lifestyle or diet [33]. At birth, Lp(a) is very low, but in the first few years of life it reaches levels similar to those in adults [34]. It is unclear whether further increases in Lp(a) levels occur throughout the growth period, so probably a single Lp(a) measurement is sufficient in children [35]. However, it is certain that children with high Lp(a) levels will become adults with similarly high levels. Furthermore, a child with high Lp(a) levels typically has at least one parent with the same condition [36]. The cardiovascular risk associated with Lp(a) is continuous, meaning there is no threshold below which the risk is absent. However, individuals with levels above 50 mg/dL (105 nmol/L) are considered to be at a higher risk; approximately 20% of the Caucasian population has levels above this threshold. Other ethnic groups, such as Black people, have higher average Lp(a) values. Nevertheless, the relationship between Lp(a) levels and cardiovascular risk can vary between ethnic groups and is influenced by other genetic and environmental factors. In the absence of specific values for the pediatric age group, the cut-offs for children are considered to be the same as those for adults [37]. A recent study performed on a population of children at cardiovascular risk showed that more than one in five children had elevated Lp(a) values. Furthermore, Lp(a) values were present in about 17% of cases in the absence of increased LDL cholesterol. These results suggest that Lp(a) measurement could help to improve the definition of the cardiovascular risk profile in children and adolescents who are already being monitored for other cardiovascular risk factors, such as high blood pressure, excess body weight and high LDL cholesterol [37]. The Italian LIPIGEN network conducted a study involving 653 children and adolescents aged 2 to 17 years who had received a clinical and/or genetic diagnosis of FH. The study found that individuals with the highest Lp(a) values were also those with a higher prevalence of early cardiovascular events among first- and second-degree relatives [38]. The occurrence of arterial ischemic stroke in infants and children has been linked to increased Lp(a) values in few studies [39,40]. However, arterial ischemic stroke is a very rare condition in childhood.

### 4.2. Hypertriglyceridemia

Although TGs do not accumulate directly in the arterial wall, TG-rich lipoproteins—especially VLDL- and chylomicron-derived remnants—promote atherosclerosis by transporting cholesterol and triggering endothelial dysfunction and vascular inflammation [41]. For this reason, hypertriglyceridemia, particularly when accompanied by other abnormalities in the lipid profile, is also considered to be a potentially atherogenic condition. However, when TG levels are very high, the primary clinical risk is not cardiovascular, but the possible development of acute pancreatitis [42].

An increase in plasma TGs occurs when there are quantitative or functional alterations to the lipoproteins responsible for transporting them, particularly chylomicrons, VLDLs and IDLs. In adults, TG values are considered normal up to 150 mg/dL, while in children they are considered normal up to 100 or 130 mg/dL for those under or over 10 years of age, respectively. Hypertriglyceridemia is considered moderately elevated up to 500 mg/dL, and is defined as very high above this value. A risk of acute pancreatitis is present when TG levels exceed 880 mg/dL (1000 mmol/L) [8]. In most cases, moderate hypertriglyceridemia is secondary to diabetes mellitus, or associated with obesity and poor eating habits, particularly alcohol abuse. If hypertriglyceridemia is associated with a reduction in HDL cholesterol, it falls within the category of atherogenic dyslipidemia, which will be discussed in detail in the section on mixed dyslipidemia. Conversely, very high TG values suggest a primary genetic alteration in TG metabolism with a polygenic or, more rarely, monogenic origin.

#### 4.2.1. Monogenic or Familial Chylomicronemia (FCS)

FCS is a rare and very serious form of hypertriglyceridemia. It manifests in childhood with potentially fatal acute pancreatitis, as well as abdominal pain, nausea, vomiting, hepatosplenomegaly and xanthomas. The condition affects one in 100,000 to one in 1,000,000 people [43]. It is an autosomal recessive disorder and, in 90% of cases, it is caused by biallelic variants of the LPL-encoding gene. In the remaining cases, other genes (*APOC2*, *APOA5*, *GPIHBP1*, and *LMF1*) are involved. These genes encode apolipoproteins or enzymes that collaborate with LPL in the hydrolysis of TGs contained in lipoproteins, allowing their uptake, especially in muscle and adipose tissue. Plasma TG levels typically exceed 1000 mg/dL, often reaching over 2000 mg/dL. The serum of these patients is therefore lipemic, containing very high amounts of chylomicrons even when fasting. Levels of other lipoproteins tend to be reduced [44].

#### 4.2.2. Polygenic Chylomicronemia

Polygenic chylomicronemia is a more common condition than FCS, occurring at a rate of one in 6000, and usually presenting in adulthood [45]. In contrast to FCS, LPL activity is not compromised by a single key gene defect, but rather by the sum of many minor alterations that produce similar effects on TG hydrolysis. While the clinical picture may resemble that of FCS, it is generally less severe. TG values tend to be high but only exceed 1000 mg/dL in the presence of precipitating factors such as alcohol abuse, overeating, uncontrolled diabetes, or other metabolic stresses. All of these factors can cause a sharp increase in TG levels and trigger acute pancreatitis. As it is a polygenic condition, polygenic chylomicronemia does not follow a Mendelian pattern of transmission, although it does show familial recurrence.

### 4.3. Mixed Dyslipidemia

#### 4.3.1. Combined Familial Hyperlipidemia

Combined familial hyperlipidemia is a common familial dyslipidemia with a complex, non-Mendelian genetic etiology. While the molecular defect is not unique, the disease is likely to be linked to a combination of genetic variants that cause increased hepatic production of apoB-containing lipoproteins [46]. Patients with this condition present with hypercholesterolemia and/or hypertriglyceridemia, which can fluctuate over the course of an individual’s lifetime and within their family. It is almost never found in children, and is rarely found in adolescents; it usually only manifests itself in adulthood. In the rare cases where it does occur in young people, the initial presentation is typically hypertriglyceridemia. Therefore, it is not included in the differential diagnosis of pediatric hypercholesterolemia.

#### 4.3.2. Atherogenic Dyslipidemia

Atherogenic dyslipidemia is a combination of dyslipidemia characterized by increased TG levels and decreased HDL cholesterol levels. While LDL cholesterol is not necessarily elevated, small, dense LDL particles are present [47]. This dyslipidemia is typical of metabolic syndrome and is usually associated with increased insulin resistance. The high cardiovascular risk mainly derives from the combination of hypertriglyceridemia, reduced HDL cholesterol and small, dense LDL particles, which exacerbate the atherosclerotic process in the context of insulin resistance and chronic inflammation. Atherogenic dyslipidemia is common in severely overweight children and adolescents, often alongside other components of metabolic syndrome such as high blood pressure and disorders of carbohydrate metabolism.

#### 4.3.3. Lysosomal Acid Lipase Deficiency (LAL-D)

This disorder is very rare, affecting only one in 90,000 to 170,000 people, but it deserves attention because those affected can benefit from specific treatment. It is a lysosomal storage disorder caused by a defect in the LAL enzyme. In this condition, cholesterol esters and TGs carried by lipoproteins are not adequately hydrolysed by enzymes once they are internalized in lysosomes via the LDLR, and so they accumulate within the cells [48]. The disease may present with an isolated increase in cholesterol levels, but it is more commonly associated with a mixed lipid profile characterized by hypercholesterolemia and variable levels of hypertriglyceridemia and reduced HDL cholesterol. LAL-D is always associated with varying degrees of liver involvement, which can manifest as hepatomegaly, steatosis, fibrosis and even cirrhosis. This should always prompt a diagnosis to be considered. The disease can present in severe forms as early as childhood [49], or in more moderate forms that may remain undiagnosed until adulthood [50].

### 4.4. Hypolipidemias

Hypolipidemias are a heterogeneous group of diseases characterized by a reduction in one or more classes of plasma lipoprotein. Only those involving a reduction in HDL cholesterol can be defined as atherogenic. Conversely, forms involving a reduction in LDL cholesterol, despite being associated with serious metabolic and clinical alterations, are not atherogenic and will therefore not be treated.

#### Hypoalphalipoproteinemia

Given the anti-atherosclerotic properties of HDL, a decrease in its levels may increase cardiovascular risk, although this is difficult to determine. Certain genetic forms caused by variants of genes such as *ABCA1*, *LCAT* and *APOA1* alter HDL synthesis or functionality [51]. A homozygous mutation in the *ABCA1* gene is the cause of Tangier disease, which is extremely rare (<1 in 1,000,000 cases). It manifests in childhood with significant decreases in HDL cholesterol levels, and the clinical presentation varies greatly from patient to patient in terms of both severity and complexity, with possible cardiovascular involvement [52]. In most cases, reductions in HDL are modest and associated with obesity, sedentary behaviour, smoking, alcohol consumption, and an unhealthy diet. Currently, there are no specific drug therapies aimed at raising HDL cholesterol levels. The main therapeutic approach is to make lifestyle changes, particularly to increase physical activity.

## 5. Diagnosis

Diagnosing atherogenic dyslipidemia in children is challenging and not fully standardized. It lies at the intersection of adult-derived biochemical criteria, developmental metabolic variability and limited long-term outcome data. The condition is characterized by a range of lipid abnormalities that traditional lipid panels do not fully capture. Although fasting lipid measurements are still the mainstay of assessment, emerging evidence suggests that advanced lipid profiling may offer additional prognostic value [53]. Despite these parameters being available, there is still uncertainty about which markers most accurately identify children at increased future cardiovascular risk. There is also uncertainty about how to account for age- and puberty-related physiological changes and how to integrate these findings into clinical decision-making [54]. Furthermore, the thresholds for intervention, particularly for children without a family history of FH, are still being debated. This reflects the wider gap between biochemical detection and evidence-based management.

### 5.1. Differential Diagnosis Between Heterozygous Familial Hypercholesterolemia and Common Polygenic Hypercholesterolemia

Genetic testing enables a definitive diagnosis of FH when a causative genetic defect is identified. If this does not apply or testing is unavailable, certain criteria can be used as a guide: age, family history, and LDL cholesterol levels. A very high LDL cholesterol value in a young child almost always suggests a familial form of the condition, as the polygenic form takes time to manifest itself. FH typically recurs in families through autosomal co-dominant transmission, meaning elevated cholesterol levels must be present in one of the parents and are frequently found in uncles or cousins on the side of the affected parent, regardless of gender. Reconstructing the family tree and confirming early cardiovascular disease and relatives’ cholesterol levels is often decisive for diagnosing FH. Several clinical scores have been developed to diagnose FH in adults. Some of these, such as the Simon Broome Register and the Make Early Diagnosis to Prevent Early Deaths (MEDPED) criteria, have also been adapted for use in pediatric subjects [55]. However, many of the criteria, particularly the presence of clinical manifestations and cardiovascular events, are absent in children [56]. Therefore, diagnosis in children is based on persistently high LDL cholesterol levels, family history, and genetic testing.

Even in the case of a polygenic form, familial recurrence may be present as families share both genetics and environment. In these cases, however, the distribution of the disease is not as precisely defined as that characteristic of FH. A cholesterol level alone is insufficient for a diagnosis, although very high levels are more indicative of a familial form. However, it is not possible to distinguish between HeFH with residual receptor activity and a polygenic form caused by a cluster of altered genes in combination with an unhealthy lifestyle and dietary habits based on cholesterol levels alone.

### 5.2. When and If to Test Plasma Lipids in Children

During childhood and adolescence, physiological fluctuations in plasma lipid concentrations occur [9]. At the beginning of puberty, cholesterol levels decrease, but by the end, they tend to return to pre-pubertal levels. Recognizing this physiological pattern is important for accurate data interpretation and should not be mistaken for an improvement if elevated cholesterol levels were previously observed. Cholesterol is essential for myelination, membrane formation, and the synthesis of hormones, vitamins, and bile acids. Therefore, limiting its intake is not recommended until the age of two, except in special cases. Therefore, the best time to measure plasma lipids is after the age of two and before the onset of puberty. Fasting is not necessary for measuring cholesterol and apolipoproteins, while TG values are higher after meals. LDL cholesterol can be measured directly in the laboratory or calculated using the Friedewald formula: LDL cholesterol = total cholesterol—(HDL cholesterol + 1/5 TG) if TG is less than 400 mg/dL. Non-HDL cholesterol is calculated by subtracting HDL cholesterol from total cholesterol. This index is important because it represents the atherogenic fraction of cholesterol, but it is not yet widely used in clinical practice. Its significance is comparable to that of apoB-100 measurement.

A much-debated issue is whether to screen all children for hypercholesterolemia or FH, or to target only those considered at risk. Undoubtedly, general screening at ages 10 and 18, as proposed by the American Academy of Pediatrics [57], would reveal a considerable number of cases of polygenic hypercholesterolemia. In most cases, these forms could be effectively treated through dietary and behavioural intervention alone, without the need for drugs. Screening would also identify individuals suspected of having HeFH. These individuals should be evaluated by expert medical staff and, in some cases, undergo genetic diagnosis and possibly receive pharmacological treatment. Although generalized screening is expensive, it should be noted that early drug treatment for HeFH would extend the life expectancy of affected children and adolescents by around twenty years, essentially eliminating their cardiovascular risk [58]. Widespread screening could be beneficial for the community if we consider the consumption of healthcare resources and years of work lost in the event of cardiovascular events. However, not everyone agrees [59]. As an alternative to general screening, another method could involve screening individuals with a positive family history and subsequently testing their relatives. The goal here would be to diagnose FH and not all forms of hypercholesterolemia. However, this targeted screening method could carry the risk of failing to identify a large number of affected individuals, given that fewer than 30% of people in the general population know their cholesterol level [60].

## 6. Treatment

For children, a non-pharmacological treatment plan involving lifestyle and dietary changes is crucial. This is because it can be effective in treating mild and moderate cases, which are generally associated with being excess weight. It also allows for lower doses of medication to be administered, even when drug therapy is necessary. Drug therapy is indicated when lipid values remain significantly elevated despite adequate dietary and behavioural treatment having been continued for a sufficient period to verify its effectiveness, or when cardiovascular risk is particularly high, as in the case of FH. According to European guidelines, drug therapy, typically involving statins, can be initiated in children with HeFH from the age of 8–10 years if they have persistently high LDL cholesterol levels. The recommended therapeutic goals are to reduce LDL cholesterol by at least 50% from baseline values and, where possible, to achieve levels below 135 mg/dL [61]. For more severe forms such as HoFH or rare genetic dyslipidemias, drug therapy should be initiated early and intensified, often involving a combination of medications, with the aim of achieving the greatest possible reduction in LDL cholesterol levels. In all cases, a personalized, progressive therapeutic approach must form part of a comprehensive care plan for children, considering factors such as age, the severity of dyslipidemia, the presence of comorbidities and the family context.

### 6.1. Non-Pharmacological Treatment of Dyslipidemia

The first studies investigating the efficacy and safety of dietary interventions for treating hypercholesterolemia in children date back to the 1980s and 1990s. The Dietary Intervention Study in Children (DISC) demonstrated that a low-saturated fat diet effectively reduces LDL cholesterol without hindering a child’s growth or pubertal development [62]. The National Cholesterol Education Programme (NCEP) released its first recommendations for managing hypercholesterolemia in children in 1992 [63]. The guidelines, which will be published later, combine dietary treatment with lifestyle intervention [8]. The first line of treatment for pediatric dyslipidemia is an intervention based on lifestyle changes and correcting eating habits, and this approach is supported by a solid body of scientific evidence. The two aims of nutritional therapy for children with dyslipidemia are to improve the lipid profile and reduce future cardiovascular risk, while ensuring an adequate nutrient intake for growth and physiological development. The effectiveness of dietary and behavioural treatment depends on several factors, including how early treatment begins, family compliance, the duration of treatment and the type of dyslipidemia being treated, since monogenic forms respond less well than polygenic forms. Studies have shown that, in HeFH, early dietary treatment can delay the onset of cardiovascular events, even if it is not sufficient to bring cholesterol levels back to normal [64].

#### 6.1.1. The CHILD-1 Diet

The Cardiovascular Health Integrated Lifestyle Diet-1 (CHILD-1) is a nutritional approach to pediatric dyslipidemia. It is recommended for children over the age of one with dyslipidemia and/or a family history of early cardiovascular disease, obesity, dyslipidemia, diabetes mellitus, primary hypertension or exposure to second-hand smoke. The CHILD-1 diet is divided into five age groups from birth to 21 years, recognizing that nutritional needs change during growth. For children aged 1 to 3 years, the diet recommends a fat intake of 30–40% of total calories to ensure adequate neurological development and myelination. For children with a family history of heart disease and hypercholesterolemia, partially fat-free milk can be introduced from 12 months of age, provided that at least 30% of their daily calorie intake comes from fat. The main features of the CHILD-1 diet for older children and adolescents are as follows:

- Total fat intake: 25–30% of total daily energy;
- Saturated fat: <10% of total energy;
- Dietary cholesterol: <300 mg/day;
- Eliminate trans fats completely;
- Favour unsaturated fats, including both monounsaturated and polyunsaturated fats.

The CHILD-1 diet involves eating nutrient-rich foods and avoiding sweets, while strictly limiting the consumption of sugary drinks. Consumption of 100% fruit juice should be limited to 120 mL (approximately 4 ounces) or less per day, with water being the main beverage. A wide variety of vegetables, fruits, lean meats and complex carbohydrates should be introduced wherever possible [8]. In the CHILD-1 diet, the amount of dietary cholesterol consumed is fixed. However, for children, it would be more appropriate to recommend an intake proportional to their daily calorie consumption. For example, 100 mg of dietary cholesterol for every 1000 calories in their diet.

#### 6.1.2. The CHILD-2 Diet

If the CHILD-1 diet does not achieve the desired plasma lipid targets within 3–6 months, switching to the CHILD-2 diet is recommended. This nutritional strategy is reserved for patients aged 2–21 years and involves more stringent restrictions than the CHILD-1 diet:

- Saturated fats: <7% of total energy intake;
- Dietary cholesterol: <200 mg/day;
- Increase dietary fibre intake, particularly soluble fibre;
- Consider taking supplements containing plant phytosterols/stanols (2 g/day);
- Strong encouragement to consume foods that are naturally rich in cholesterol-lowering substances.

#### 6.1.3. The Mediterranean Diet

Studies have shown that the Mediterranean diet can effectively improve the lipid profile in children with dyslipidemia. The greater the adherence to the diet, the better the results [65]. The main recommendations of the Mediterranean diet adapted for the pediatric age group are as follows:

- Eat at least five portions of fresh fruit and vegetables per day. As well as providing vitamins, minerals and fibre, these foods contain polyphenols and carotenoids, which have antioxidant and anti-inflammatory properties.
- Whole grains and legumes should be the main sources of complex carbohydrates. Legumes provide vegetable protein, soluble fibre and micronutrients, which contribute to glycemic control and improve the lipid profile.
- Fish should be eaten at least two to three times a week as the main source of animal protein. Oily fish such as mackerel, sardines, anchovies and salmon are rich in long-chain omega-3 fatty acids (eicosapentaenoic acid and docosahexaenoic acid), which have beneficial effects on LDL composition.
- Nuts and oilseeds (such as walnuts, almonds, hazelnuts, flax seeds and chia seeds) should be consumed in moderation due to their high calorie content. However, they are a source of unsaturated fats, fibre, vegetable proteins and micronutrients.
- Use extra virgin olive oil as the main fat for seasoning. It is rich in oleic acid and phenolic compounds with antioxidant and anti-inflammatory properties and is associated with beneficial effects on the lipid profile and endothelial function.
- Consume fermented dairy products, mainly yoghurt, in moderation.
- Red and processed meats should be consumed in very limited quantities and replaced by lean white meats.

It is also essential to eliminate the consumption of simple sugars, particularly in liquid form. The glucose and, more importantly, the fructose contained in soft drinks stimulates de novo lipogenesis and the production of uric acid, as well as other intermediate compounds that have harmful metabolic effects. Furthermore, by stimulating insulin production, they promote the development of metabolic syndrome and non-alcoholic fatty liver disease [66,67].

#### 6.1.4. Physical Activity

Regular physical activity is an essential part of the treatment for dyslipidemia, even in children, due to its multiple beneficial effects. Aerobic exercise increases HDL cholesterol levels by stimulating LPL activity and apoA-I synthesis. Additionally, exercise reduces TG levels by increasing the use of fatty acids as an energy source during exercise and improving insulin sensitivity afterwards. For children with atherogenic dyslipidemia and insulin resistance, another important aspect is that exercise improves muscle glucose uptake through insulin-independent mechanisms mediated by muscle contraction. This contributes to glycemic control and reduces hyperinsulinemia. Furthermore, regular physical activity reduces visceral fat, which contributes to insulin resistance by secreting pro-inflammatory adipokines and free fatty acids [68,69].

According to international recommendations, children and adolescents should engage in at least 60 min of moderate-to-vigorous physical activity per day, predominantly aerobic. They should also engage in muscle- and bone-strengthening activities at least three times a week. It is also important to reduce the time spent on sedentary activities, particularly screen time (TV, video games, smartphones and tablets), to no more than 2 h per day [70,71].

#### 6.1.5. Dietary Supplements

Although dietary supplements that claim to lower cholesterol and improve lipid profiles are often used in children, this report has chosen not to discuss them due to their unproven safety and efficacy in this age group. Conversely, foods containing added plant sterols, which compete with cholesterol absorption, may be useful in treating children with hypercholesterolemia.

### 6.2. Drug Treatment of Dyslipidemia

The following drugs are currently available for use in children. Those currently approved for use in children for the treatment of hypercholesterolemia include bile acid binding agents, statins and ezetimibe. These represent the basis of therapy for the most common forms, particularly FH. In addition, there are drugs available for treating rare and severe forms of dyslipidemia, which can be used in specialist centres [72]. In the most severe cases that do not respond to drug therapy, lipoprotein apheresis may be recommended. This extracorporeal procedure allows LDL to be selectively removed from plasma, and is a fundamental therapeutic option for patients with HoFH from a pediatric age onwards [73].

#### 6.2.1. Bile Acid Binding Agents (Cholestyramine)

These drugs decrease the recovery of bile through the enterohepatic circulation, thereby promoting the elimination of cholesterol, one of the main components of bile. For a long time, bile acid binding agents were the only pharmacological treatment option for pediatric hypercholesterolemia. However, their lipid-lowering efficacy is modest, with an average change in LDL cholesterol levels of around −17% in children with FH [74]. Currently, statins are the preferred option, with cholestyramine use is limited to cases where statins cannot be used due to intolerance (which is rare in children) or in subjects younger than eight to ten years of age. In adults, in more severe cases, cholestyramine may be prescribed alongside other lipid-lowering drugs [75]. Although cholestyramine is an effective drug, it has the major limitation of poor palatability, which is why many children refuse to take it. It must be taken before the two main meals of the day, at a dosage of at least 4 g. Due to its mechanism of action, cholestyramine can interfere with the absorption of fat-soluble vitamins and folates [76]. Therefore, their levels must be monitored and supplemented if necessary.

#### 6.2.2. Statins

Statins work by inhibiting the action of hydroxymethylglutaryl-Coenzyme-A (HMG-CoA) reductase, the key enzyme involved in cholesterol production in the liver. Reduced cholesterol synthesis increases demand for this substrate, leading to two effects: on the one hand, LDLR is upregulated, increasing the liver’s uptake of circulating cholesterol; and on the other hand, intestinal absorption of dietary cholesterol increases. As the second effect is smaller than the first, the net result is a decrease in cholesterol levels.

Depending on the type and dosage of statin used, these drugs cause a 20–50% decrease in cholesterol levels compared to baseline values [77]. Several pediatric guidelines suggest the use of these drugs, with restrictions based on age, LDL cholesterol levels, and the presence of other cardiovascular conditions or risk factors. A Cochrane review, which included nine randomized and controlled clinical studies involving 1177 children and adolescents with HeFH, concluded that statin treatment is an effective short-term lipid-lowering therapy (median follow-up: 24 weeks) [7]. A more recent review confirmed that statin therapy significantly reduces LDL cholesterol levels in pediatric patients with FH, with minimal adverse effects [78]. Moreover, initiating statin therapy during childhood in patients with FH slows the progression of vascular damage and reduces the risk of cardiovascular disease in young adulthood suggesting a potential long-term reduction in cardiovascular risk [5].

Table 3 shows the statins that can be used in children, with indications for their prescription.

The use of statins is permitted from 8 to 10 years of age, after 3–6 months of dietary and behavioural treatment, in the presence of LDL cholesterol ≥ 190 mg/dL, or ≥160 mg/dL with a positive family history in first- or second-degree relatives for early cardiovascular disease or other cardiovascular risk factors, or with LDL cholesterol ≥ 130 mg/dL and the presence of multiple and serious cardiovascular risk factors.

Statins are generally well tolerated by children. The most clinically relevant adverse effects are myalgia and a possible, generally modest and reversible, increase in transaminases. In most studies, there were no significant differences in adverse events compared to the placebo group. Statins did not have an adverse effect on growth or pubertal development [5,79]. There have been no reported cases of liver failure or rhabdomyolysis related to statin therapy in children. However, periodic monitoring of transaminases and creatine phosphokinase is recommended during therapy. Statins should be taken orally, preferably in the evening.

#### 6.2.3. Ezetimibe

Ezetimibe is the most recently made available drug for treating hypercholesterolemia in children. It inhibits the absorption of cholesterol and phytosterols (but not triglycerides, fatty acids, bile, steroid hormones or fat-soluble vitamins) in the duodenum and small intestine by binding to the sterol transporter (Niemann-Pick C1-Like 1 protein) located on the brush border of enterocytes. Ezetimibe is fairly effective on its own (reducing cholesterol levels by about 20%) [80], and is mainly used as monotherapy when other cholesterol-lowering drugs are not tolerated. In a small sample of children with PH and FH ezetimibe significantly lowered total cholesterol and LDL cholesterol [80]. A review of a small retrospective series of children and adolescents treated with ezetimibe for hypercholesterolemia confirmed that this drug is safe and effective at lowering LDL cholesterol levels [81]. Finally, an RCT enrolling 138 children aged 6–10 with HeFH or clinically relevant non-FH showed that ezetimibe led to significant reductions in LDL cholesterol, with a favourable safety profile [82]. Although this drug works well alongside statins by reducing the absorption of dietary cholesterol that statins tend to increase, combining statins and ezetimibe in the same tablet is not recommended for children. Ezetimibe is taken orally as a single 10 mg dose at any time of day, regardless of meals, from the age of 10 in the US. The drug is generally well tolerated.

#### 6.2.4. Anti-PCSK9 Therapies

These therapies target PCSK9 and represent one of the most significant innovations in the treatment of hypercholesterolemia, particularly severe genetic forms. The two anti-PCSK9 monoclonal antibodies currently available—evolocumab [83] and alirocumab [84]—bind to the circulating protein, thereby preventing interaction with the LDL receptor. Numerous randomized clinical trials in adults have shown that these drugs can reduce LDL cholesterol levels by approximately 50–60% when used alongside standard statin and ezetimibe therapy, thereby reducing the risk of major cardiovascular events. Some studies conducted in pediatric patients suggest that these drugs are as effective as those observed in adults, particularly in patients with HeFH. The HAUSER-RCT study showed that evolocumab reduced LDL cholesterol levels by 44.5% (75 mg/dL) in a group of 104 children and adolescents (aged 10–17) with HeFH, compared to the placebo group (*n* = 53), over a 20-week follow-up period [83]. A significant reduction of −35.3% compared to baseline was also observed after 80 weeks of follow-up, with no major adverse events reported [85]. A pooled data analysis of three studies in which evolocumab was used to treat a small number of pediatric patients with HoFH revealed significant variability in response to the drug with regard to LDL cholesterol levels. Nevertheless, 42.9% of patients achieved an LDL cholesterol reduction of >15% from baseline [86]. A more recent RCT comparing alirocumab to a placebo in 153 patients aged 8–17 years with HeFH, demonstrated a significant reduction in LDL cholesterol in the treatment group over a 24-week follow-up period [84]. Thanks to these studies, this class of drugs should soon also be used in children. It is administered subcutaneously, using a special device, once every two weeks or once a month.

New drugs have recently been developed that inhibit hepatic PCSK9 synthesis using RNA interference. One such drug is inclisiran, which achieves a significant and sustained reduction in LDL cholesterol with very infrequent administration. However, experience with these drugs in children is currently absent or extremely limited [87], and they are only approved for use in adults. A single Phase 3 RCT (ORION-13) involving a small number of adolescents with HoFH (*n* = 13; mean age: 14.8 years) demonstrated moderate efficacy of inclisiran in reducing LDL cholesterol levels compared with placebo. However, the sample size was too small to draw definitive conclusions about the use of the drug in this age group [87].

#### 6.2.5. Evinacumab

Evinacumab is a human monoclonal antibody that targets Angiopoietin-Like Protein 3 (ANGPTL3). ANGPTL3 is a liver protein that inhibits the activity of plasma lipases involved in the metabolism of triglycerides and apoB-rich lipoproteins. Inhibiting ANGPTL3 increases the activity of lipoprotein and endothelial lipases, which reduces LDL cholesterol and TG levels independently of the LDL receptor. This characteristic makes evinacumab particularly effective in patients with HoFH, for whom treatments that increase LDL receptor expression or function are ineffective or only partially effective [88]. In clinical trials conducted on adults and adolescents with HoFH, evinacumab was shown to reduce LDL cholesterol by an average of 45–50% compared to the placebo group [89]. The drug is administered intravenously every four weeks. Recently, evinacumab was approved for use in children with the most severe forms of HoFH only [89,90,91].

#### 6.2.6. Lomitapide

Lomitapide is a drug that inhibits microsomal TG transfer protein, which is essential for the assembly of lipoproteins containing apoB (i.e., VLDL and chylomicrons), thereby reducing LDL cholesterol generation. As the cholesterol-lowering effect is independent of LDLR activity, lomitapide is effective in treating the most severe forms of HoFH [92]. The main toxicity associated with this drug is the accumulation of lipids in the liver, which can lead to steatosis and steatohepatitis. For this reason, treatment must be combined with a strict low-fat diet and careful monitoring of liver function. Lomitapide is not approved for use in children, although its off-label use in severe cases of HoFH has been reported [93].

#### 6.2.7. Bempedoic Acid

Bempedoic acid works by inhibiting Adenosine Triphosphate-citrate lyase, an enzyme involved in cholesterol synthesis that acts upstream of HMG-CoA reductase. It is a prodrug that is mainly activated in the liver by an enzyme that is minimally or not at all expressed in skeletal muscle [94]. For this reason, it is not associated with a significant increase in the risk of adverse muscle events, a known limitation of statin therapy. Bempedoic acid is not currently approved for use in children. A clinical trial assessing the pharmacokinetics, pharmacodynamics and safety of Bempedoic Acid in children aged 6 to 17 with HeFH is currently ongoing (Clinical Trials NCT05694260 [95]).

### 6.3. Treatment of Hypertriglyceridemia

The only treatment indicated for hypertriglyceridemia in children is dietary and behavioural therapy based on lifestyle changes, such as reducing the intake of simple sugars and controlling body weight. In cases of mixed dyslipidemia, which are rare in children, the main objective is to lower cholesterol levels, including through drug therapy when necessary. For rare forms of severe hypertriglyceridemia (TG values > 500–1000 mg/dL), dietary restrictions are accompanied by the administration of omega-3 fatty acids (eicosapentaenoic acid and docosahexaenoic acid) in high doses appropriate for age and weight to achieve a clinically significant effect [96]. These are the only drugs approved for pediatric use. However, a retrospective study examining the efficacy of omega-3 as an adjunct therapy for treating elevated hypertriglyceridemia in obese children (mean age 12.7 years) failed to demonstrate any additional benefit of omega-3 therapy over lifestyle modification alone in decreasing plasma TGs levels [97]. In contrast, a RCT showed a 39.1% reduction in TGs in obese children taking 3 g of omega-3 fatty acids daily, compared to a 14.6% reduction in the placebo group [98].

Although other pharmacological options are available for treating hypertriglyceridemia, they are only used in adults. Fibrates are the main class of drugs used in adults thanks to their ability to significantly reduce plasma TG levels by activating the peroxisome proliferator-activated receptor-α receptor. However, a lack of adequate efficacy and safety studies has so far prevented their approval for use in children. Recently, new drugs that target specific regulators of TG metabolism have been developed. These include antisense oligonucleotides and interfering RNAs that target apolipoprotein C-III, such as volanesorsen and olezearsen. These molecules have been shown to reduce TG levels markedly in adults with severe hypertriglyceridemia and familial chylomicronemia syndrome. Nevertheless, these drugs are not yet approved for use in children and adolescents and are only used in experimental settings or controlled clinical trials.

## 7. Conclusions

Atherogenic dyslipidemia in children and adolescents is a clinically significant yet frequently overlooked condition with significant implications for long-term cardiovascular health. Available evidence clearly indicates that early lipid abnormalities contribute to cumulative vascular damage, and that timely intervention can substantially modify lifetime risk trajectories. Lifestyle and dietary measures remain the cornerstone of management and should be universally promoted. Pharmacological treatment is only justified for selected high-risk patients, particularly those with FH or severe lipid abnormalities. Nevertheless, several challenges remain, including establishing the optimal screening strategy for pediatric populations, integrating Lp(a) measurement into clinical practice, defining treatment thresholds for different age groups, and addressing the limited long-term safety data for early pharmacological intervention. Meanwhile, the rapid development of novel lipid-lowering therapies offers promising prospects, but also raises questions regarding cost-effectiveness, accessibility and the appropriate selection of patients in pediatric settings. Addressing these issues will require well-designed longitudinal studies and closer collaboration between pediatricians, lipid specialists and primary care providers. It is essential to increase awareness and education among healthcare professionals to improve early detection, ensure evidence-based management, and ultimately reduce the burden of cardiovascular disease originating in childhood.

## Figures and Tables

**Table 1:** Reference values for plasma lipids in children.

	Acceptable mg/dL	Border Line mg/dL	High mg/dL	Low mg/dL
Total cholesterol	<170	170–199	>200	
LDL cholesterol	<110	110–129	>130	
Triglycerides < 9 years	<75	75–99	>100	
Triglycerides > 10 years	<90	90–129	>130	
HDL cholesterol	>45	35–45		<35
ApoB-100	<90	90–109	>100	
ApoA-I	>120	115–120		<115
Lipoprotein(a)	<30	30–50	>50	

HDL = high-density lipoprotein; LDL = low-density lipoprotein. Modified from National Institutes of Health National Heart, Lung, and Blood Institute 2011 [8].

**Table 2:** Main secondary dyslipidemias.

Form of Dyslipidemia	Cause
Hypercholesterolemia	Obstructive liver disease Medications: progestins, cyclosporine, thiazides Hypothyroidism Acute intermittent porphyria Nephrotic syndrome
Hypertriglyceridemia	Alcohol Diabetes mellitus Acute hepatitis Medications: estrogens, isotreonine, β-blockers Glucocorticoids, bile acid binders, thiazides Monoclonal gammopathies, multiple myeloma, lymphoma Glycogen storage disease Pregnancy Chronic renal failure Ileal bypass Lipodystrophy Systemic lupus erythematosus Sepsis Stress
HDL cholesterol reduced	Medications: β-blockers, steroids, anabolics Tobacco smoking Malnutrition due to deficiency

HDL = high-density lipoprotein.

**Table 3:** Statins that can be used in pediatric age.

Drug	Strength Pharmacological *	Dose mg/Die	Approval US FDA	Technical Data Sheet Italy
Atorvastatin	+++	10–20	>10 years	>10 years
Fluvastatin	+	20–80	>10 years	>9 years
Lovastatin	+	10–40	>10 years	age not defined
Pravastatin	+	20–40	>8 years	from 8 years 8–13 years 10–20 mg/die >13 years 10–40 mg/die
Rosuvastatin	++++	5–20	>10 years	from 6 years 6–9 years 5–10 mg/die >10 years 5–20 mg/die
Simvastatin	++	10–40	>10 years	>10 years males Tanner pubertal stage > II, females 1year post-menarche

FDA = Food and Drug Administration. * The column expresses the average potency of the statin in reducing LDL cholesterol [61].

## Data Availability

No new data were created or analyzed in this study.

## Abbreviations

The following abbreviations are used in this manuscript:

ANGPTL3	Angiopoietin-Like Protein 3
Apo B-48	apoprotein B-48
Apo B-100	apoprotein B-100
Apo A-I	apoprotein A-I
CHILD-1	Cardiovascular Health Integrated Lifestyle Diet-1
CHILD-2	Cardiovascular Health Integrated Lifestyle Diet-2
C	Cholesterol
FCS	Monogenic or familial chylomicronemia
HDL	high-density lipoprotein
HeFH	heterozygous
HMG-CoA	hydroxymethylglutaryl-Coenzyme-A
HoFH	homozygous familial hypercholesterolemia
IDL	intermediate density lipoprotein
LAL	lysosomal acid lipase
LAL-D	lysosomal acid lipase deficiency
LDL	low-density lipoprotein
LDLR	low-density lipoprotein receptor
Lp(a)	lipoprotein(a)
LPL	lipoprotein lipase
PCSK9	proprotein convertase subtilisin/kexin type 9
TG	triglyceride
VLDL	very low-density lipoprotein
